# A Case of Acute Autoimmune Hepatitis Superimposed on Chronic Hepatitis B Infection

**DOI:** 10.1155/2018/2139607

**Published:** 2018-04-01

**Authors:** Vanessa Sostre, Hiren G. Patel, Abdalla Mohamed, Ariy Volfson

**Affiliations:** ^1^Department of Internal Medicine, St. Joseph's University Medical Center, Paterson, NJ, USA; ^2^Department of Gastroenterology and Hepatology, St. Joseph's University Medical Center, Paterson, NJ, USA

## Abstract

Autoimmune hepatitis has been associated with chronic HCV infection, but there are only few cases reported of HBV infection as a possible trigger. We present a case of a young male who was diagnosed with acute autoimmune hepatitis superimposed on existent chronic HBV infection. A 30-year-old Hispanic male with no past medical history presented to the hospital with complaints of few days of generalized weakness. Laboratory findings were significant for elevated liver enzymes: AST, 1164 U/L; ALT, 1461 U/L; total bilirubin, 2 MG/DL; and alkaline phosphatase, 75 IU/L. Extensive workup was done to find the etiology for elevated liver enzymes. Only blood work that came back positive was for chronic HBV infection and elevated immunoglobulin G (IgG) level 1937 mg/dL. HBV viral load was 42,900,000 IU/mL. The patient was started on tenofovir 300 mg daily. Liver biopsy was done which was consistent with autoimmune hepatitis. Prednisone 60 mg daily was started. Six months later, blood work showed completely normal liver enzymes and total IgG. Hepatotropic viruses have been proposed as triggering factors for several autoimmune diseases. There are theories suggesting that similarity in viral epitope and self-proteins expression on liver cells' surface causes a cross-reactive immunologic response and possible viral-induced autoimmune hepatitis.

## 1. Introduction

Autoimmune hepatitis (AIH) is an uncommon chronic liver inflammation with an unclear etiology [1]. Like most of the autoimmune diseases, it is predominant in females with a prevalence of less than 0.02% [1]. “Lupoid hepatitis,” as it was called in the past, has been associated with other hepatic diseases such as drug-induced liver injury, primary biliary cholangitis, primary sclerosing cholangitis, and viral hepatitis, specifically hepatitis C virus [2]. There are few cases reported for hepatitis B virus (HBV) as a possible trigger of this rare disease [3–7]. We present a case of a young male who was diagnosed with acute AIH superimposed on underlying chronic HBV infection.

## 2. Case Report

**Figure 1:**
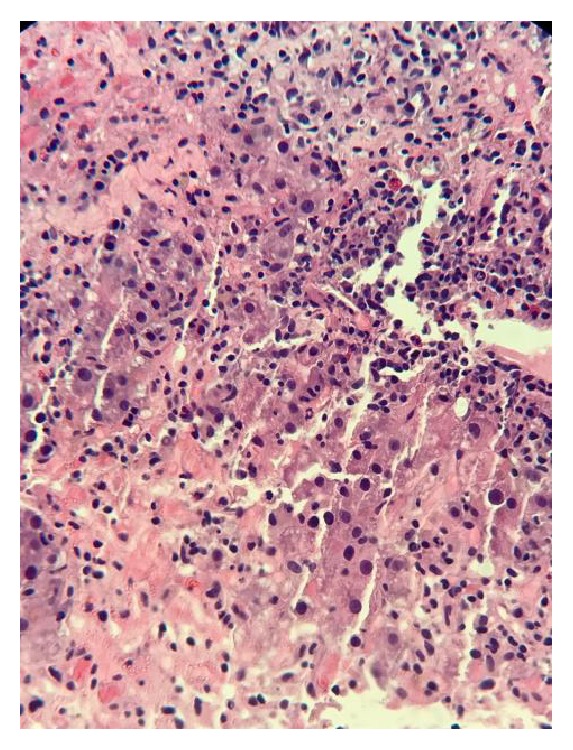
H&E stain of liver biopsy at high power.* Liver Biopsy Pathology Report*. Prominent lymphoplasmacytic infiltrate in the portal tracts with marked interface activity and multiple areas of hepatic necrosis. In some foci, plasma cells are particularly prominent.

**Figure 2:**
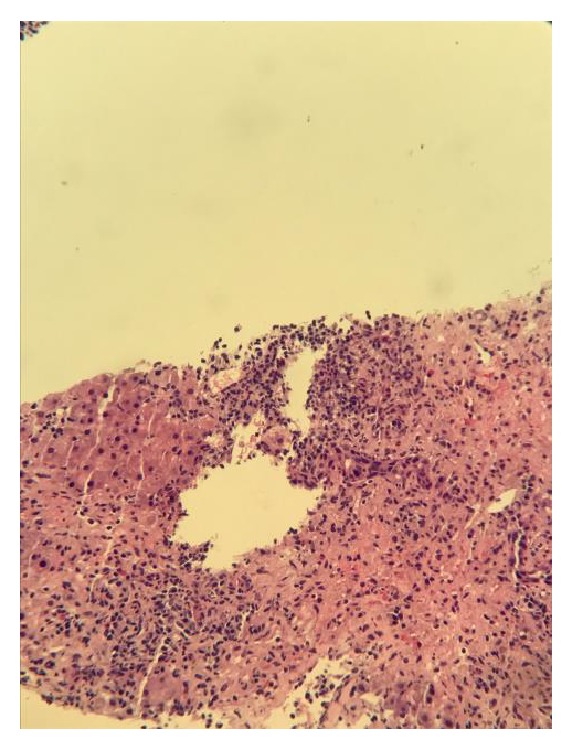
H&E stain of liver biopsy at low power.* Liver Biopsy Pathology Report*. Prominent lymphoplasmacytic infiltrate in the portal tracts with marked interface activity and multiple areas of hepatic necrosis. In some foci, plasma cells are particularly prominent.

A 30-year-old Hispanic male with no past medical history presented to the hospital with a complaint of generalized weakness for a few days. The patient denied abdominal pain, nausea, vomiting, pruritus, illicit drug use, skin tattoos, blood transfusions, alcohol abuse, acetaminophen use, recent travel, or multiple sexual partners. He denied any history of liver disease. Physical exam was completely normal. Laboratory findings were significant for elevated liver enzymes: AST, 1164 U/L; ALT, 1461 U/L; total bilirubin, 2 MG/DL; alkaline phosphatase, 75 IU/L; PT/INR, 14.5/1.1. An extensive workup was done to find the etiology of elevated liver enzymes. Only blood work that came back positive was for chronic Hep B infection (positive for HBsAg, HBeAg, and HBcIgG; negative for HBsAb and HBcIgM) and elevated total immunoglobulin G (IgG) level 1937 mg/dL. The rest of the workup including acetaminophen level, hepatitis C antibody, HAV Ab IgM, hepatitis D Ab, EBV DNA, HSV DNA, CMV DNA, and hepatitis E Ab came back negative. The autoantibodies for AIH including ANA, ASMA, and anti-LKM also came back negative. Ultrasound of the liver was unremarkable. Hepatitis B viral load was 42,900,000 IU/mL. The patient was started on tenofovir 300 mg daily. Liver biopsy was done, which demonstrated lymphoplasmacytic infiltrate with prominent plasma cells in the portal tracts with marked interface activity and multiple areas of hepatic necrosis consistent with autoimmune hepatitis (Figures 1 and 2). Three days after starting tenofovir, no significant improvement in liver enzymes was seen, so prednisone 60 mg once a day was started. 48 hours after starting prednisone, liver enzymes level dropped significantly (Table 1). The patient was discharged home on tenofovir 300 mg daily and prednisone 60 mg daily. One week after discharge, the patient was seen in the outpatient clinic and at that time transaminases and IgG level were significantly trended down (Table 1). The patient was given a tapering dose of prednisone 40 mg/day on week 2 and 30 mg/day on weeks 3 and 4 and was kept on 20 mg/day as a maintenance dose. The patient was on tenofovir and maintenance dose of prednisone at 6-month follow-up. Blood work showed completely normal liver enzymes and total IgG with undetected HBV viral load (Table 1). Autoantibodies for AIH were still negative in the blood work which was done at 6-month follow-up.

## 3. Discussion

Hepatotropic viruses have been proposed as triggering factors for several autoimmune diseases; for example, chronic HCV infection has been associated with the development of AIH [2]. Pathogenesis of development of AIH in patients with chronic HBV infection is not well understood. There are theories suggesting that the target site for an immunologic response might be related to viral antigens being expressed on liver cells' surface [3]. Molecular mimicry defined as similarity in viral epitope and self-proteins causes a cross-reactive immunologic response that could cause the development of viral-induced autoimmune hepatitis [2, 4, 8]. When there is no active HBV replication, low suppressor T-cell activity was demonstrated in cases with AIH. This is not completely proven, but previous studies suggested that this process allows B-cells to proliferate and develop autoantibodies to liver cells causing this autoimmune phenomenon [3]. The stimulation of antigen presenting cells or expression of major histocompatibility complex on the cell membranes by inflammatory cytokines allowed the risk of autoreactivity during the response to viral infection [9].

In order to diagnose AIH, it is important to exclude other causes of the chronic liver disease first. Patients with AIH usually have elevated liver enzymes and IgG. High levels of IgG have been seen in up to 85% of patients [2]. There are three types of AIH based on serologic findings. AIH type 1 is the most common with elevated anti-smooth muscle antibodies (SMA) and antinuclear antibodies (ANA). Elevated levels of anti-liver kidney microsome 1 (anti-LKM-1) and anti-liver cytosol type 1 antibody (anti-LC-1) are present in AIH type 2. Two markers for AIH type 3 are anti-soluble liver antigen (anti-SLA) and anti-liver-pancreas (anti-LP). The presence of these autoantibodies is important but not pathognomonic for the diagnosis of AIH [2]. These autoantibodies are absent in about 10% of AIH patients [10]. Seronegative AIH was described both in adults and in children with the same demographic, biochemical, and histologic features of classical AIH but negative autoimmune serology [11, 12]. As per the clinical practice guideline for AIH, seronegative patients at diagnosis should be monitored for serologic markers as autoantibody titers may fluctuate and can be expressed later during the course of the disease [2]. There are many studies reporting that some of the patients previously diagnosed with cryptogenic chronic active hepatitis probably had seronegative AIH as they had clinical remission after prednisone therapy and avoided progression to end-stage liver disease [13–15]. Responsiveness to immunosuppressive therapy supports the autoimmune phenomena in all previously reported cases [4]. Although serological markers for AIH were negative in our patient, a finding based on liver biopsy, rapid improvements of transaminases and IgG level were highly suggesting seronegative AIH on chronic HBV infection.

We do not know for sure whether chronic HBV infection was the trigger factor for the development of AIH in our case. More research would require understanding the complicated pathophysiology of this rare viral-induced autoimmune disease.

## Figures and Tables

**Table 1:** Trend of laboratory results.

Labs	Labs on presentation	Day 1 of prednisone	Day 2 of prednisone	Day 3 of prednisone	Day 10 of prednisone, one-week follow-up	6-month follow-up
AST (U/L)	1164	1258	855	562	155	81
ALT (U/L)	1461	2010	1676	1265	430	39
ALP (U/L)	75	98	87	94	121	63
T. bilirubin (MG/DL)	2	4.6	4.7	4.1	3.4	0.9
Total IgG (MG/DL)	1937	NA	1632	NA	1427	1192
PT (SEC)	14.5	19.1	19.1	17.2	14.3	13.8
INR	1.1	1.6	1.6	1.4	1.1	1
HBV (IU/MI)	42900000				2699	HBV viral load undetected
